# Clinical validation of real-time tissue change monitoring during prostate tissue ablation with high intensity focused ultrasound

**DOI:** 10.1186/s40349-017-0102-2

**Published:** 2017-09-13

**Authors:** Narendra T. Sanghvi, Wo-Hsing Chen, Roy Carlson, Clint Weis, Ralf Seip, Toyoaki Uchida, Michael Marberger

**Affiliations:** 1 SonaCare Medical, LLC, 4000 Pendleton Way, Indianapolis, IN 46226 USA; 2 Ulthera Inc., Merz Device Innovation Center, 1840 S Stapley Drive, Ste. 200, Mesa, AZ 85204 USA; 3 Hachioji Urologic Clinic, 3-6-7, Koyasucho, Hachioji, Tokyo 192-0904 Japan; 4 Medical University of Vienna, Waehringer Guertel 18-20, A-1090 Vienna, Austria

**Keywords:** High intensity focused ultrasound, Tissue change monitoring, Prostate tissue temperature, Thermocouples, Prostate tissue ablation, Hemi-ablation

## Abstract

**Background:**

The purpose of these clinical studies was to validate a Tissue Change Monitoring (TCM) algorithm in vivo. TCM is a quantitative tool for the real-time assessment of HIFU dose. TCM provides quantitative analysis of the backscatter pulse echo signals (pre and immediately post HIFU) for each individual ablative site, using ultrasonic tissue characterization as a surrogate for monitoring tissue temperature. Real-time analysis generates an energy difference parameter (ΔE in dB) that is proportional to tissue temperature.

**Methods:**

Post in vitro studies, two clinical studies were conducted to validate the TCM algorithm on the Sonablate® device. Studies enrolled histologically confirmed, organ confined prostate cancer patients. The first clinical study was conducted in two phases for whole gland ablation. First eight patients’ data were used to measure the algorithm performance followed by 89 additional patients for long term outcome. The second clinical study enrolled five patients; four patients with focal cancer had hemi-ablation only and one had whole gland ablation. Four 3 Fr. needles containing three thermocouples each were placed transperineally in the prostate to record tissue temperatures in the focal zone, posterior to the focal zone and on the lateral gland where no HIFU was applied. Tissue temperatures from the focal zone were correlated to the ΔE parameter.

**Results:**

In the first clinical study, the average TCM rate was 86%. Pre and 6 months post HIFU, median PSA was 7.64 and 0.025 ng/ml respectively and 97% patients had negative biopsy. For the second clinical study, the measured prostate tissue temperatures (Average, Max, and Min) in the ablation zones were 84°, 114° and 60 °C and the corresponding ΔE (dB/10) parameters were 1.05, 2.6 and 0.4 resulting in 83% of temperatures in the range of 75°-100 °C and 17% in the 60°-74 °C range. Outside the focal zone, the average temperature was 50 °C and in the lateral lobe where no HIFU was applied, peak temperature was 40.7 °C.

**Conclusions:**

The TCM algorithm is able to estimate tissue changes reliably during the HIFU procedure for prostate tissue ablation in real-time and can be used as a guide for HIFU dose delivery and tissue ablation control.

## Background

Since the early 1990s, ultrasound (US) image guided, transrectal high-intensity focused ultrasound (HIFU) has been extensively researched for the treatment of prostatic disease. Earlier animal and human studies found HIFU to be an effective and safe minimally invasive energy delivery modality to induce contact and ionizing radiation free intraprostatic coagulative tissue necrosis while sparing the intervening tissue [1–4]. Transrectal HIFU tissue ablation is ideally suited as a minimally invasive modality for prostatic tissue because of the proximity of the HIFU transducer to the prostate gland. This approach offers technical advantages including the ability to operate the device at higher ultrasound frequencies for both imaging and HIFU dose. The higher ultrasound frequency results in accurate prostate localization with high resolution B-mode ultrasound imaging and precise thermal lesions production at the targeted sites. Additionally, transrectal ultrasound scanning (TRUS) for imaging and biopsy guidance for prostatic tissue is an established clinical modality, hence an ultrasound guided transrectal HIFU device is a practical delivery approach for the urology clinical practice. These salient features expanded the ultrasound guided HIFU application for the ablation of localized prostate cancer [5–7]. Now there are several HIFU devices available for prostate tissue ablation, but this report specifically describes studies conducted using a modified Sonablate® device. The ultrasound image guided HIFU device, Sonablate®, displays simultaneously “pre ablation” (reference) and “during ablation” B-mode transverse and sagittal images in real-time. The simultaneous display of these images is used to both plan the ablation sites and monitor in real-time the progress of the HIFU procedure. B-mode ultrasound images display hyperechoic regions when there is a presence of cavitation or vapor bubbles when﻿ tissue temperature in the HIFU focal site is elevated to boiling levels [2]. The phenomena associated with tissue boiling have been used in prostate tissue ablation under the well known “Visually Directed HIFU procedure” [8]. However, B-mode ultrasound imaging systems do not display thermal lesions produced in the temperature range of 60–90 °C, as ultrasound images are generated from the rectified amplitude of the backscattered echo signals with limited dynamic range and contrast. In the meantime, numerous investigators in the ultrasound field have suggested temperature dependent ultrasound tissue characterization [9–17] for the purpose of HIFU and thermal therapy procedures monitoring. Many of these investigators found that among other temperature dependent ultrasound tissue parameters, backscattered echo signals energy is a highly reliable indicator for HIFU dose, as the backscattered signal energy increases significantly when tissue temperature is raised above 60 °C [9–13]. Worthington et al. investigated the effect of water bath heating on freshly excised human prostate tissue at 45 °C, 50 °C, 55 °C, 60 °C, and 65 °C with 5 MHz broadband ultrasound. In this study the ultrasound attenuation coefficient and backscattered echo signals power of fresh human prostate tissue were measured. The attenuation coefficient and backscattered echo signals power increased by factors of 2.7 and 9, respectively, during the 65 °C heating. The study concluded that these two ultrasound parameters have important implications for HIFU treatment planning and monitoring of thermal therapy. Our group [Seip et al. [18]] investigated multiple thermal lesion detecting algorithms specifically for prostate tissue ablation in an in vivo canine study. Algorithms sensitive to *relative* tissue changes during HIFU measuring backscattered echo signals energy, tissue displacement, entropy, and tissue attenuation were compared for their ability to detect the creation of multiple and adjacent thermal lesions. In vivo (*N* = 4) canine prostate backscattered pulse-echo signals were acquired with a custom Sonablate® 500 HIFU device during 7 ablative sessions. A total of 815 ablative sites were exposed to HIFU with total acoustic powers used for the production of coagulative necrosis with a 3 s ON and 6 s OFF duty cycle, forming the algorithm evaluation dataset. It was found that the algorithm based on backscattered echo signals energy performed best, detecting 82% of all thermal lesions created, while showing false positive rates below 5%. This investigation was the foundation for the Tissue Change Monitoring (TCM) algorithm based HIFU device for prostate tissue ablation presented in this report.

## Methods (Backscattering data acquisition and temperature measurement)

In this report, we present a modified Sonablate® HIFU system with a tissue change monitoring (TCM) algorithm that calculates the backscattered echo signals energy increase (ΔE in dB) for each individual HIFU ablative site and assigns green, yellow or orange color overlay on the sagittal image to represent a mild, moderate or large change induced in the ablated tissue by the HIFU dose in real-time. This color information helps the user to increase or decrease the HIFU dose by adjusting the ultrasound total acoustic power (TAP) for the subsequent sites. In addition, at the end of the procedure, the user can review all targeted ablative sites in 3D rendering and cross-sections images of the prostate gland and re-target those sites that did not show significant change by TCM. Prior to implementing this technique for routine clinical use in the HIFU device, TCM was validated both in vitro and in vivo studies including multiple thermocouples implanted in the prostate gland to monitor tissue temperature.

**Fig. 1:**
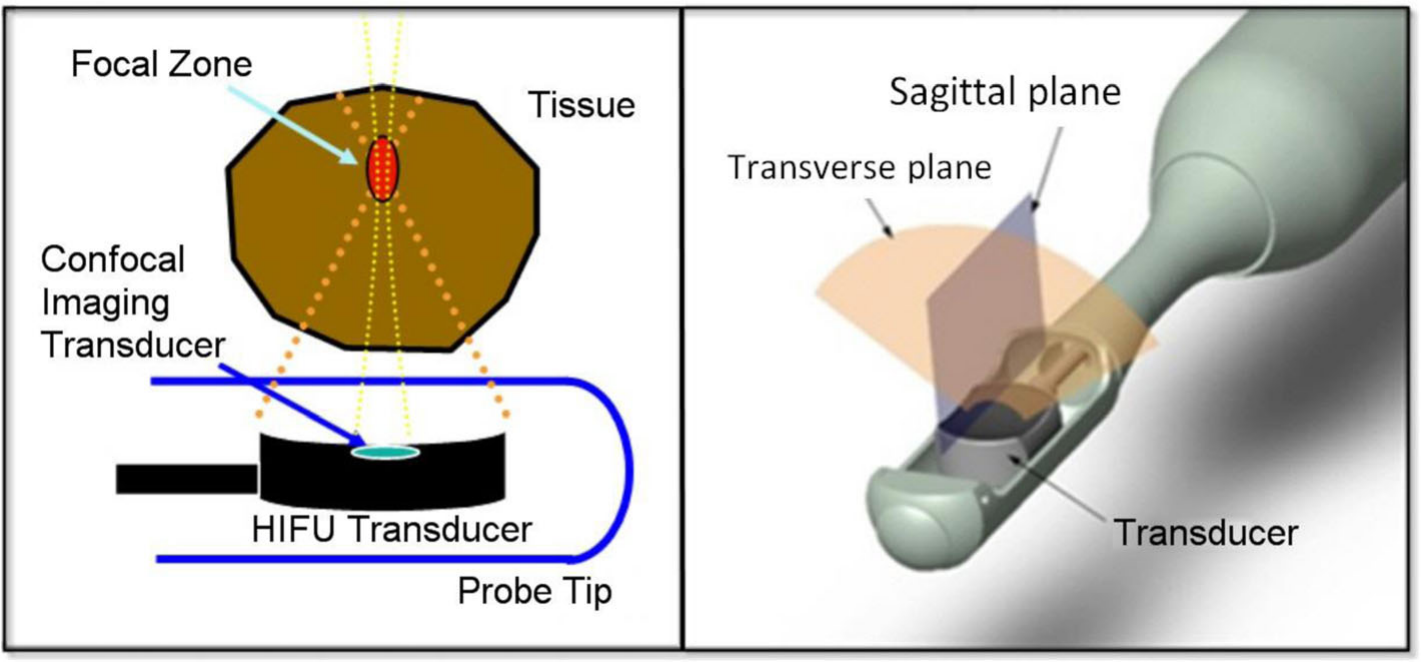
The Sonablate® probe tip with a confocal imaging and HIFU transducer. The transducer assembly contains 3.0 cm and 4.0 cm focal lengths HIFU transducers on opposite sides

The Sonablate HIFU system has been described in detail previously [19] and is briefly described here. The transrectal probe has a confocal ultrasound imaging and HIFU therapy transducer assembly as illustrated in Fig. 1. The center element of the transducer is used for pulse-echo ultrasound imaging of the tissue and the outer element is used to deliver the HIFU dose. The transducer assembly contains 3.0 cm and 4.0 cm focal length (Fl) HIFU transducers on opposite sides. For ablation of anterior and middle zones of the prostate, the 4.0 cm Fl is used and for the ablation of posterior zone tissue the 3.0 cm Fl is used.

**Fig. 2:**
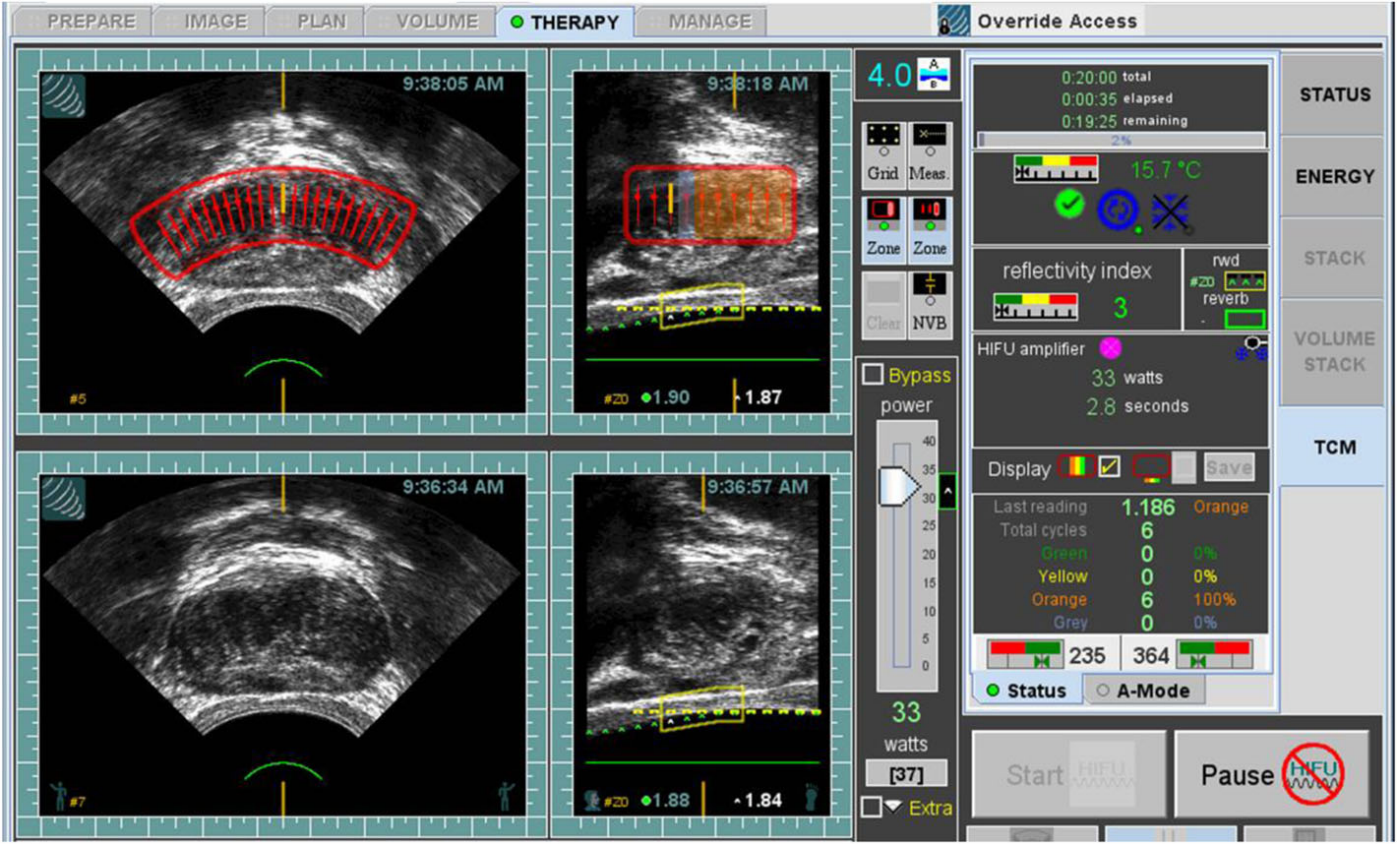
The Sonablate 500 TCM monitor display during the whole gland ablation of prostate tissue. The lower (reference) and upper (real-time) panels are transverse and sagittal ultrasound images of the prostate (pre and post HIFU) for each ablative site, respectively. The upper panel images are marked with transducer focal zone and selected tissue ablative sites. The ΔE calculation is computed after each HIFU exposure and a green, yellow or orange color is overlaid on the ablated site as per Table 1 color scheme

This unique transducer configuration provides accurate spatial registration of prostate and surrounding tissue for imaging, planning and ablation. The computer controlled automated probe images the entire prostate in both sagittal (XZ) and transverse (YZ) planes by mechanically scanning the ultrasound transducer as shown in Fig. 2. These B-mode images are orthogonal and spatially register all pixels via the ultrasound velocity parameter in tissue. Both images are used to localize and plan for prostate tissue ablation. The 2D ultrasound images are rendered by processing the backscattered echo signals of high frequency sound waves from the tissue. The backscattered echo signals containing phase and amplitude information are digitized at a 50 MHz sampling frequency with a 12 bit analog to digital converter. For ultrasound imaging purposes, the phase information of the backscattered signals is discarded, and the amplitude information is envelop and peak detected to display in a gray-scale image, with a dynamic range that is compressed (−24 dB) to meet the display characteristics. For TCM purposes, both the phase and amplitude information of the backscattered signals are used in the signal processing algorithm to derive an energy difference parameter (ΔE in dB). To obtain more accurate TCM estimates and to minimize errors in the data acquisition due to motion and/or swelling of the prostate gland, pre and post HIFU backscattered echo signals are acquired rapidly (< 6 s) for each ablative site. For TCM analysis, 15 consecutive pulse–echo backscattered echo signals (lines) are used from each ablated site. The distance between each pulse-echo line is 0.2 mm. These 15 backscattered echo signals (lines) cover the entire ablation site, as consecutive ablation sites are spaced 3 mm apart from each other in the sagittal plane.

### In vitro test

Temperature vs. Spectral energy - (ΔE change vs. Tissue Temperature and TCM Color Mapping scheme).

**Fig. 3:**
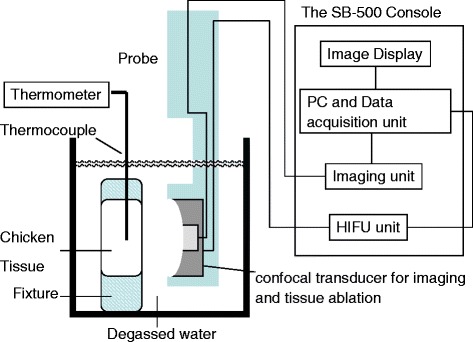
The Sonablate system setup block diagram for in vitro test

**Fig. 4:**
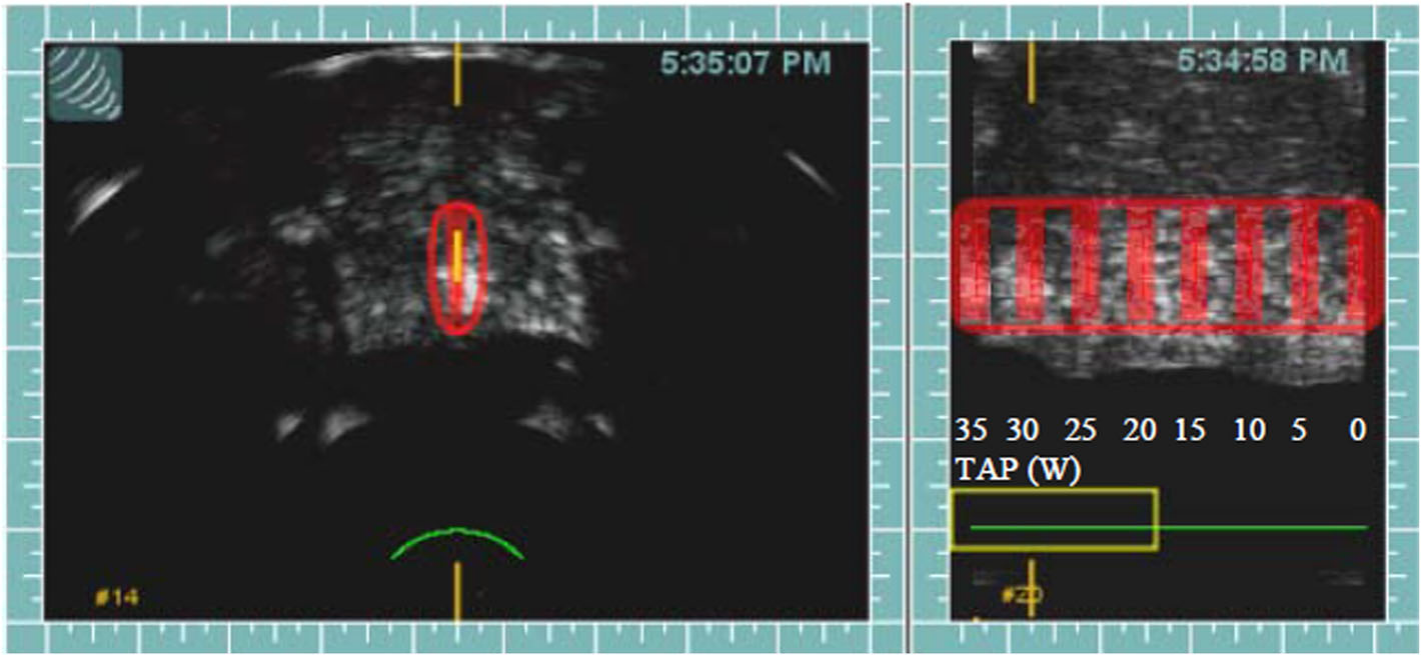
Sonablate HIFU ablation planning on chicken tissue (before TCM color coding). The left and right panel images are B-mode ultrasound transverse and sagittal image of the tissue. Each red color box is a HIFU ablation site. The HIFU ablation started from the right and the transducer was translated to the left in 6 mm increments, and the acoustic power was increased by 5 watts for each subsequent exposure

The modified Sonablate HIFU system with TCM software was tested with freshly excised chicken breast tissue in vitro. The chicken breast tissue was pre-heated to 30 °C and submerged in degassed water at room temperature. The chicken tissue was imaged and the device gain settings were adjusted to keep backscattered pulse-echo signals within the 12 bit digitization dynamic range to avoid saturation. As shown in Fig. 3, the probe was placed in front of the tissue and a HIFU tissue ablation was planned using sagittal and transverse B-mode images of the tissue as shown in Fig. 4. The 3 mm ablation sites were separated by 6 mm to provide delineation of each individual thermal lesion in the tissue. The HIFU cycle was 3 s ON and 6 s OFF. The first HIFU ablation site was located at the right most side on the sagittal image. The initial applied HIFU power was 0 watts and increased to 35 W by 5 W increaments for each subsequent site as the transducer was stepped by 6 mm to the left. This power settings allowed to test clinically relevant TAP levels of 20 to 37 W to ablate 30–35 cm^3^ prostate tissue. Typically these power levels generate 1500–2200 W/cm^2^ in-situ focal intensity and create an individual lesion of @ 10–12 mm long in the axial beam direction.

Temperature at each ablative site was measured using a thermometer (HH506RA, Omega Engineering, Inc.) and type T, copper-constantan, isonel-insulated, 0.002″ diameter thermocouple (Physitemp Instruments, Inc., Clinton, NJ). The insertion and location of the thermocouple tip in the HIFU focal zone was accurately guided under the sagittal and transverse imaging.

With these settings, the in vitro tests were repeated several times. Pre- and post HIFU backscattered pulse-echo signals and peak temperature at each site were recorded for data analysis.

### Data analysis and color scheme

The TCM signal processing algorithms are described in detail by Chen et al. [20, 21] and briefly presented below. The backscattered pulse-echo signals are analyzed in the frequency domain using the Fast Fourier Transform (FFT) to derive the ΔE value for each ablative site as described by eqs. 1, 2 and 3. For real-time analysis, a 6 MHz bandwidth (2–8 MHz) was used that matched to the electro-acoustic characteristics of the imaging element and imaging receiver amplifier. The data processing was limited to a two dimensional (12 mm × 3 mm) region of interest (ROI) that spatially matched the focal zone of the HIFU transducer dimensions. A maximum value of ΔE was derived from lower (2–3 MHz), middle (3–4.8 MHz) and upper (5–8 MHz) frequency bands [20].

$$ \mathrm{SR}\ \left(\ \mathrm{f}\ \right)=20\ \log 10\mid \mathrm{FFT}\left\{\mathrm{Hann}\ \left[\mathrm{sr}\ \left(\mathrm{t}\right)\ \right]\right\}\mid \kern1.25em (1). $$

$$ \mathrm{SL}\ \left(\ \mathrm{f}\ \right)=20\ \log 10\mid \mathrm{FFT}\left\{\mathrm{Hann}\ \left[\mathrm{sl}\ \left(\mathrm{t}\right)\ \right]\right\}\mid \kern1.25em (2). $$

$$ \varDelta \mathrm{E}\ \left(\mathrm{f}\right)=\mathrm{SL}\ \left(\mathrm{f}\right)\hbox{-} \mathrm{SR}\ \left(\mathrm{f}\right);\mathrm{magnitude}\  \mathrm{difference}\  \mathrm{of}\ \mathrm{pre}-\mathrm{and}\ \mathrm{post}-\mathrm{HIFU}\  \mathrm{in}\ \mathrm{dB}\kern0.5em (3). $$

where f = Frequency in MHz, Hann = Hanning window of 256 points for FFT analysis,

sr (t) = pre-HIFU pulse-echo signal and sl (t) = post-HIFU pulse-echo signal.

Color Scheme: Table 1 shows a color mapping scheme that was developed to represent a range of temperatures and corresponding values for ΔE computed from the in vitro studies. These colors are overlaid on the B-mode for each ablated site based on the ΔE value.

**Table 1:** TCM Color Mapping for Tissue Temperature range and corresponding increased ΔE

TCM Color	Measured Peak Temperature (°C)	The Range of the Temperature Increase ΔT (°C)	ΔE (dB)
	48	<18	<3.3
	65	19–35	3.3–6.6
	90	36–60	>6.6

### Clinical validation

This TCM based HIFU device was validated in two clinical studies. The first TCM software validation study was performed at the Department of Urology, Tokai University Hachioji Hospital, Hachioji, Japan. The objectives of this study were: A) to define the ability of the algorithm to calculate ΔE in real-time and to display color mapping on the corresponding ultrasound images during HIFU ablation of prostate tissue; and B) to define tissue ablation efficacy by following patients with prostate tissue biopsy and nadir prostate specific antigen (PSA (ng/ml)) at 6 months. The second TCM algorithm validation was conducted at the Department of Urology, University of Vienna, Vienna, Austria. The objective of this study was to correlate peak temperatures in vivo and calculated ΔE values for each ablative site in prostate cancer patients.

### Materials and methods of the TCM software validation clinical study

**Fig. 5:**
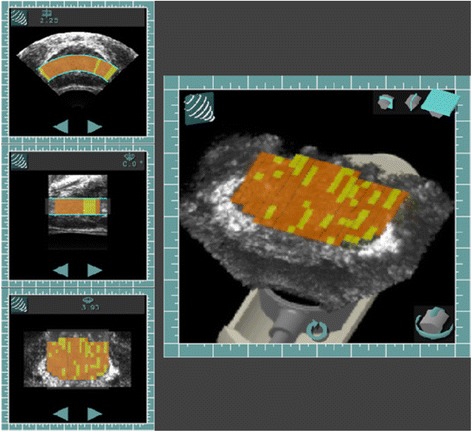
3D rendering of whole gland ablated prostate. The left panel show transvers, sagittal and coronal images with the TCM color scheme for each ablated site. The left is a magnified view of the coronal image showing some sites in green. The user can re-target any of these sites for ablation

Eight histologically confirmed organ confined prostate cancer patients were enrolled as per the clinical study protocol [22] with intent to ablate the entire prostate gland with the TCM software based Sonablate 500 device [21, 23]. All patients were under local epidural anesthesia for the HIFU procedure. The whole prostate gland ablation sites were divided in 2 or 3 focal zones based on the prostate’s anterior-posterior (AP) dimension. A 4.0 cm focal length transducer was used to plan and ablate the prostate’s anterior and middle zones, and a 3.0 cm focal length transducer was used to plan and ablate the prostate’s posterior zone. Under computer control, the transducer was mechanically scanned in both transverse and sagittal planes to plan the ablation. Multiple parallel transvers images (at 3 mm step size) of the prostate from the base to apex of the gland were obtained to select the ablative sites. For each selected ablation site in the transverse plane, prostate tissue was ablated from the apex to the base in 3 mm increments in the sagittal plane as shown in Fig. 2. The total acoustic power (TAP) level was adjusted by the operator based on the tissue depth between the rectal-wall and the focal zone using the TAP slider on the monitor. At the completion of whole gland ablation, the prostate was imaged in multiple parallel transverse planes from the base to the apex with a step size of 0.5 mm. These images were used to render 3D volumetric image of the whole prostate gland (Fig. 5). This 3D imaging feature also allowed the imaging of the prostate in transverse, sagittal and coronal planes at all locations (sites) in the prostate. Each ablative site location was registered in this 3D rendering and was overlaid with designated TCM colors. This feature allowed the operator to select a site and ablate it again where it was found a sub-optimal ablation, generally a site overlaid by green color. For the first eight patients in the study, each patients’ pulse-echo (backscattered RF) data was analyzed and then TCM success detection rate for each prostate was calculated as: (number of ablative sites having ΔE > 3.3 dB/total number of ablative sites). Within 24 h post HIFU ablation, the prostate was imaged with a T1 weighted MR sequence, 1 min following gadolinium contrast agent, to estimate the regions of coagulative tissue necrosis. The prostatic specific antigen (PSA) for each patient was recorded pre and post HIFU at 3, 6 and 12-month intervals. The 6 month follow up also included a random prostate biopsy. This device and procedure was then followed for an additional 89 patients (with the exception of MR imaging of the prostate).

### Material and methods of In-Vivo temperature correlation to ΔE clinical study

**Fig. 6:**
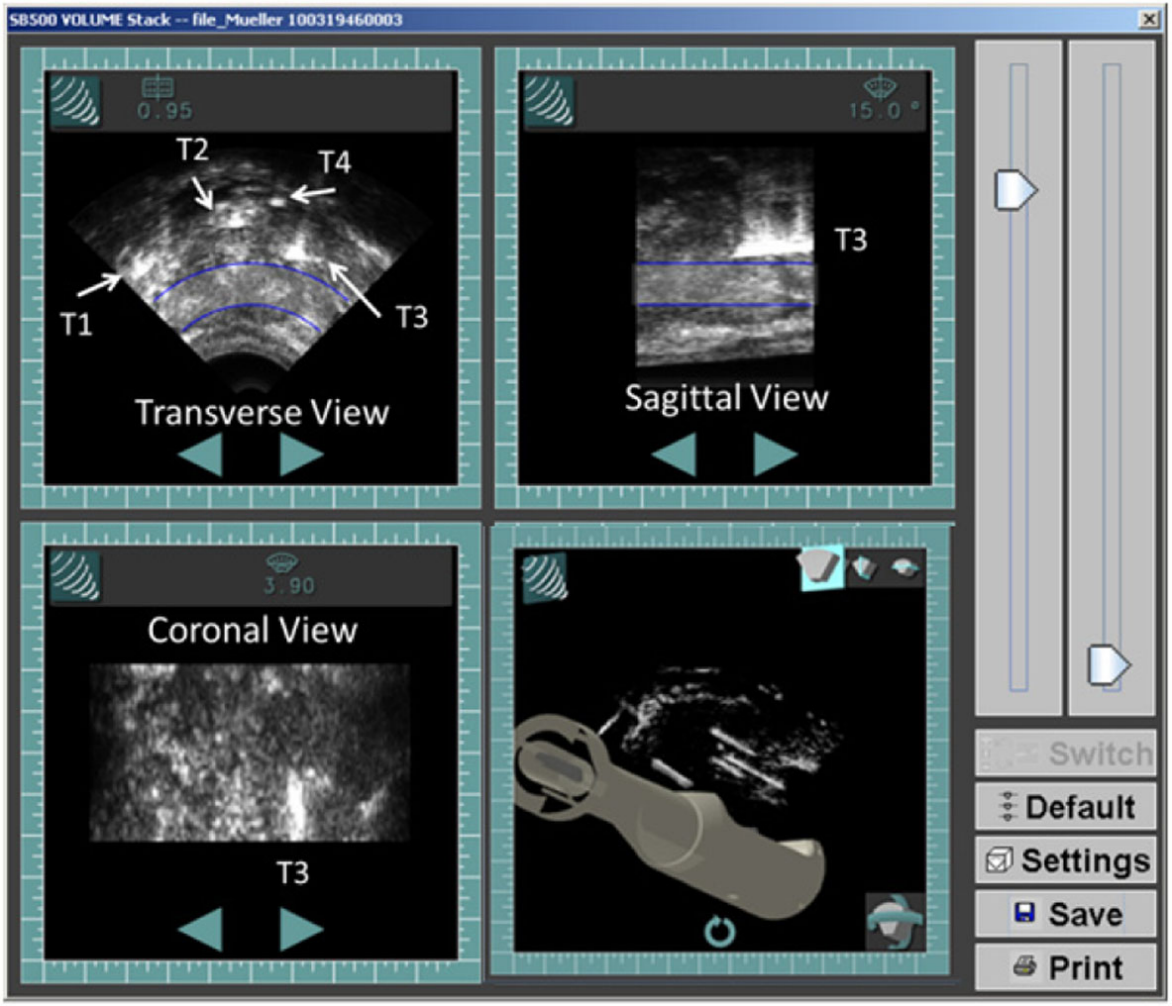
All four needle tips are identified by arrows in the transverse plane (T1, T2, T3 and T4). The sagittal and coronal images shows T3 needle entering from apex to middle of the prostate gland. All four needles were visible in 3D rendering

Five patients with histologically confirmed, organ confined prostate cancer were enrolled with the institutional review board (IRB) approved clinical protocol for this clinical study. Four patients with focal cancer at stereotactic saturation biopsy had hemi-ablation only and one had a whole gland ablation. All patients were under general anesthesia for the procedure. The Sonablate 500 HIFU device with Tissue Change Monitoring (TCM) algorithm was used for the prostate ablation. Thermocouple needles and temperature recording during HIFU in vivo procedure are described in detail by Sanghvi et al. [2]. Briefly, four needles (3 Fr.), each containing three thermocouples (type T, diameter 60–100 m, manufactured by Physitemp Instruments, Inc., Clinton, NJ) separated by a 1 cm distance inside the cannula were placed transperineally under TRUS guidance inside the prostate as depicted in Fig. 6. For all patients, at least one needle was placed inside the focal zone. The needles were identified on the ultrasound images as bright echoes. Temperatures from all thermocouples were simultaneously recorded at a 0.5 s sampling interval by a 16 channel digital temperature recording device LT-100 (Labthermics, Champaign, IL). The LT-100 device has a temperature measuring range of 0–120 °C and an accuracy of +/− 0.1 °C. The LT-100 and Sonablate clocks were synchronized during the study for offline data analysis. Temperature data was corrected to account for thermal conduction and ultrasound reflection by the needle. This correction factor was derived in our laboratory by exposing thermocouple needle and a bare wire (60–100 μm) thermocouple in the Sonablate HIFU focal field at various acoustic power levels. The transient peak temperature difference between the bare wire and needle thermocouples is dependent on the surrounding tissue temperature and ultrasound intensity. For the surrounding temperature of 37 °C, the noted temperature difference between the bare wire and needle thermocouple was @15 °C for applied acoustic power of 35 W. During the steady state conditions (such as hot water) there is negligible temperature difference between these two thermocouples. For each patient, the calculated ΔE value for each individual ablative site was matched with the recoded temperature from a thermocouple nearest to that site.

**Fig. 7:**
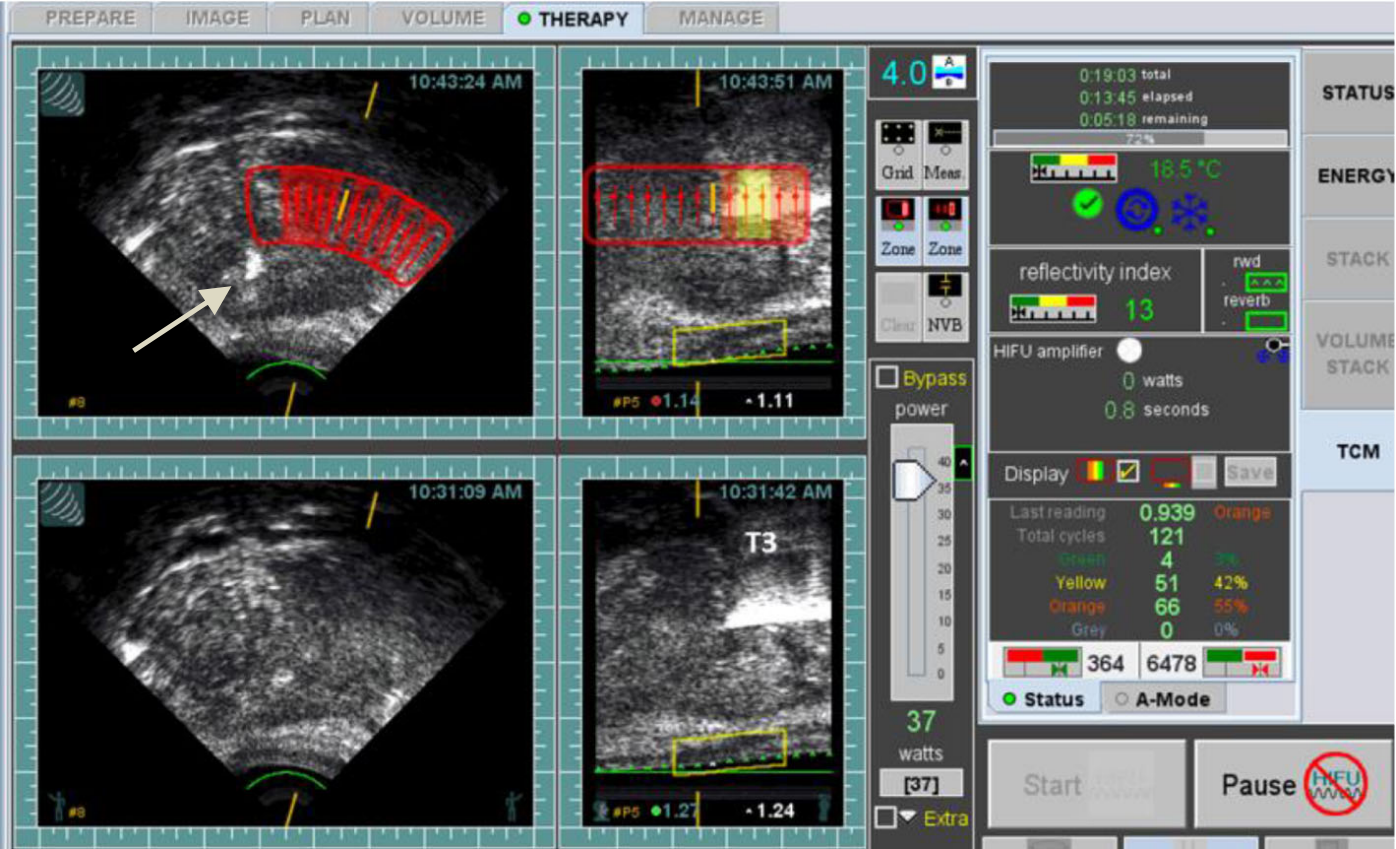
Hemiablation of the prostate. The upper panel transverse image shows the HIFU ablation targeted only for the left half of the gland. The lower panel images are reference images taken prior to tissue ablation process. The prostate is ablated with a 4 cm focal length transducer with TAP of 37 watts. Thermocouple needle T3 is displayed as a hyperechoic line within the focal zone of the HIFU transducer. Another thermocouple needle is outside the focal zone (seen on the transverse image-marked with an arrow). The upper panel images are rendered immediately post HIFU ablation for each site. The TCM color is overlaid on the site as per ΔE value at the site

Based on saturation biopsy results, four patents had hemi-ablation of the prostate gland (Fig. 7) and one patient had a whole gland ablation. All prostates were ablated using 3 focal zones. The anterior and middle zones were ablated with a 4.0 cm focal length transducer and posterior zone was ablated with a 3.0 cm focal length transducer.

The average total acoustic power (TAP) used for ablation was 37, 35 and 19 W for the anterior, middle and posterior zones respectively.

## Results

### In vitro study results

**Fig. 8:**
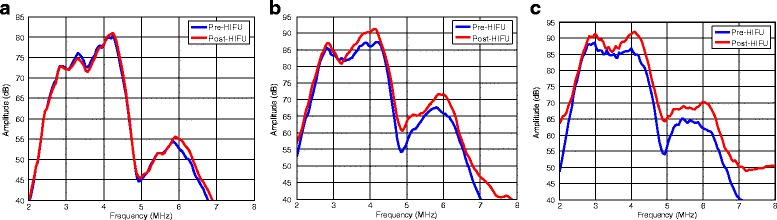
Examples of the Pre-HIFU (blue) and Post-HIFU (red) power spectra and corresponding color map for total acoustic powers of 5 W, 20 W and 30 W, respectively. **a** TCM Green (TAP = 5 W), (**b**) TCM Yellow (TAP = 20 W), (**c**) TCM Orange (TAP = 30 W)

Typical frequency spectra for pre and post HIFU of chicken tissue are shown in Fig. 8 for 5, 20 and 30 watts TAP levels. The magnitude difference between the pre and post spectra increased with increased TAP. A noticeable increase in ΔE (magnitudes) was observed in the lower frequency band and upper frequency band for TAP values above 25 W with temperature readings over 65 °C.

**Fig. 9:**
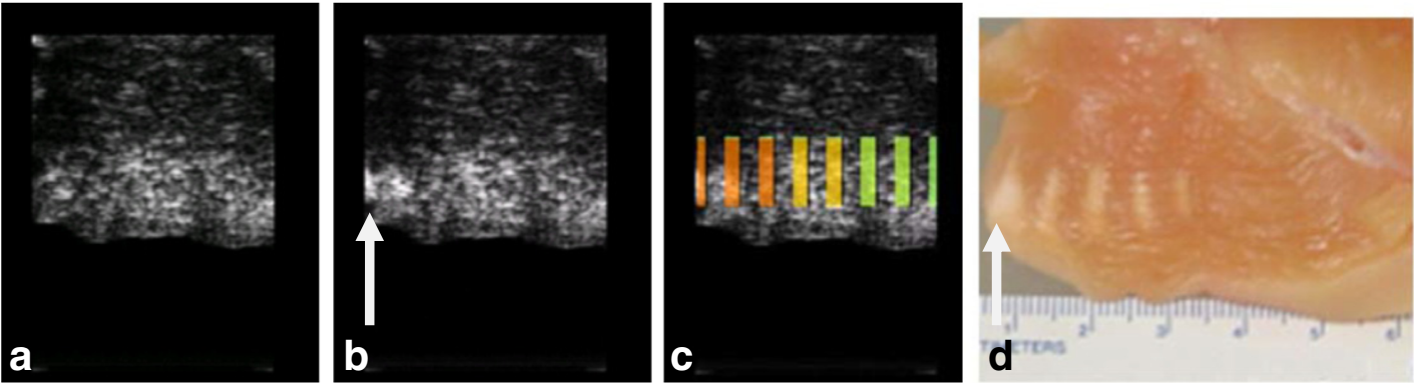
**a** Pre-HIFU sagittal image. **b** Post-HIFU sagittal image. **c** Post-HIFU sagittal image with TCM color overlay (after color calibration). The overlay color is based on ΔE magnitude as per Table 1. **d** Freshly excised chicken tissue cross-section in the plane of HIFU ablation. Thermal lesion in the tissue is visible as a blanched region. The size and shape of the blanching corresponds to a TAP value at that site. Thermal lesion was not visible for TAP values less than 15 watts

A small echogenic region was noticed in the B-mode (Fig. 9 - panel (b)) with an arrow when the TAP levels were 30 and 35 watts. The visible individual thermal lesions were developed when the acoustic power level were above 15 watts. The lesion size (approximately 10 mm × 3 mm) and shape were closer to the expected thermal lesions for 25–30 watts. At 35 watts the lesion was similar to a tadpole shape (panel (d) highlighted by arrow) and produced hyper echogenicity in the B-mode.

**Fig. 10:**
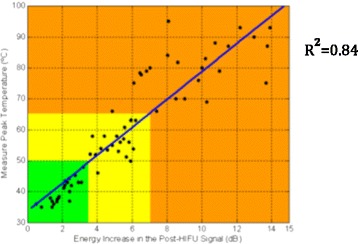
Peak tissue temperature Vs. Increased Energy (ΔE) of HIFU ablated chicken tissue in-vitro tests (*n* = 4) with color scheme of Table 1. The trend line and data-set had a cross correlation of 0.84

These in vitro tests (*n* = 4) generated a dataset for temperature vs. ΔE (dB) values (Fig. 10) and thermal lesions size and shape observed on sliced tissues. The linear cross correlation coefficient between a trend line and the dataset was 0.84. Thermal lesions were matched with the temperature readings. There were no thermal lesions seen for temperatures below 48 °C. For the peak temperatures in the range of 48 °C and 65 °C, lesions were visible but pale and smaller than 10 mm × 3 mm, and for the higher temperature range of 65–95 °C lesions were pronounced with expected size. Green, Yellow and Orange colors for Table 1 were selected to represent no thermal lesion, small but visible thermal lesion and fully developed thermal lesion of the expected size respectively.

### TCM software validation study results

Prior to the clinical study, pulse-echo data was collected with a pre-TCM version of the Sonablate device. This dataset allowed us to perform off-line data analysis and sort out the ablated sites generating the backscattered signal energy increase (ΔE) above 3.3 dB.

An eight patient study using the new TCM calculations showed the thermal lesion detection rates (defined as - number of sites with ΔE > 3.3 dB/total number of ablated sites in the whole prostate gland) in Table 2, with pre and post HIFU nadir PSA at 6 months. All eight patients had negative prostate biopsy at 6 months.

**Table 2:** TCM Success Rates and PSA post HIFU tissue ablation

Patient No.	1	2	3	4	5	6	7	8
Total Ablative Sites	508	868	1168	680	848	674	679	646
Success rate % (ΔE > 3.3 dB)	78	90	92	93	73	86	90	86
Clinical Outcome								
Pre-PSA (ml/ng)	6.28	8.9	7.33	13.63	10.0	7.95	4.92	6.5
Six months Post HIFU nadir PSA (ng/ml)	0.04	0.02	0.11	0.01	0.03	0.03	0.01	0.01

**Fig. 11:**
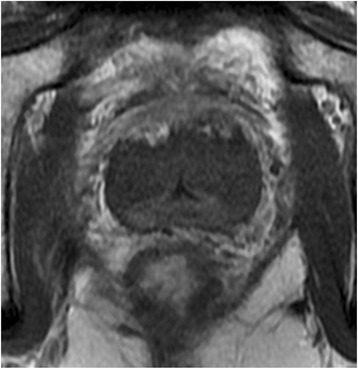
T1 W tranvers MRI, 1 min post Gadolinium contrast agent. The lack of contrast agent uptake is indicative of tissue coagulation

All eight patients had T1 W Gadolinium contrast enhanced MRI of the pelvic that showed tissue necrotic regions (Fig. 11).

Based on this eight patients’ results, the study continued to gather data for additional 89 patients with the Sonablate device with TCM. For these patients, power levels were guided by TCM generated color codes. These patients did not have MRI follow-up. All patients PSA data are summarized in Table 3.

**Table 3:** PSA Results

Clinical Follow up with additional 89 patients	Pre-HIFU	Post-HIFU (@6 months)
Total Number of patients (*N*)	97 (8 + 89)	97
Min PSA (ng/ml)	0.7	0.0
Max PSA (ng/ml)	76	4.33
Median PSA (ng/ml)	6.89	0.07
Standard Deviation (SD)	11.19	0.62

The post HIFU negative biopsy rate for this group of patients was 97%.

### In-Vivo temperature correlation to ΔE clinical study results

Thermocouple readings during the prostate HIFU ablation provided a comparison of TCM results to temperature.

**Fig. 12:**
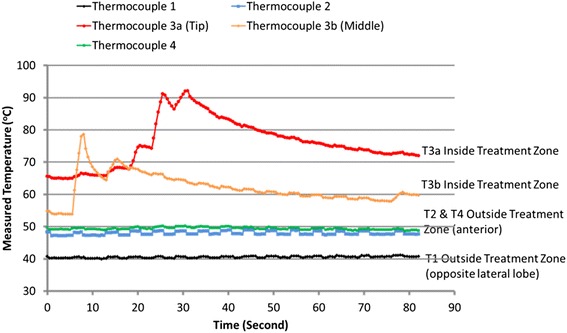
Recorded prostate tissue temperatures at various locations inside the gland during HIFU ablation. Thermocouple T3a is at the tip of the needle situated very close to the HIFU focal spot and T3b is 1 cm distal (toward apex of the prostate)

The measured temperatures (Average, Max, and Min) in the HIFU ablation zones were 84, 114 and 70 °C respectively. TCM energy readings (Average, Max, and Min) were 1.05, 2.6 and 0.4 (dB/10), resulting in 83% orange (75–100 °C) and 17% yellow (60–74 °C) sites, indicating an estimated average temperature of 91 °C. Outside the focal zone, average recorded temperature was 50 °C. The temperature recorded in the lateral lobe where no HIFU was applied was 40.7 °C. Typical prostate tissue temperatures are shown in Fig. 12.

**Fig. 13:**
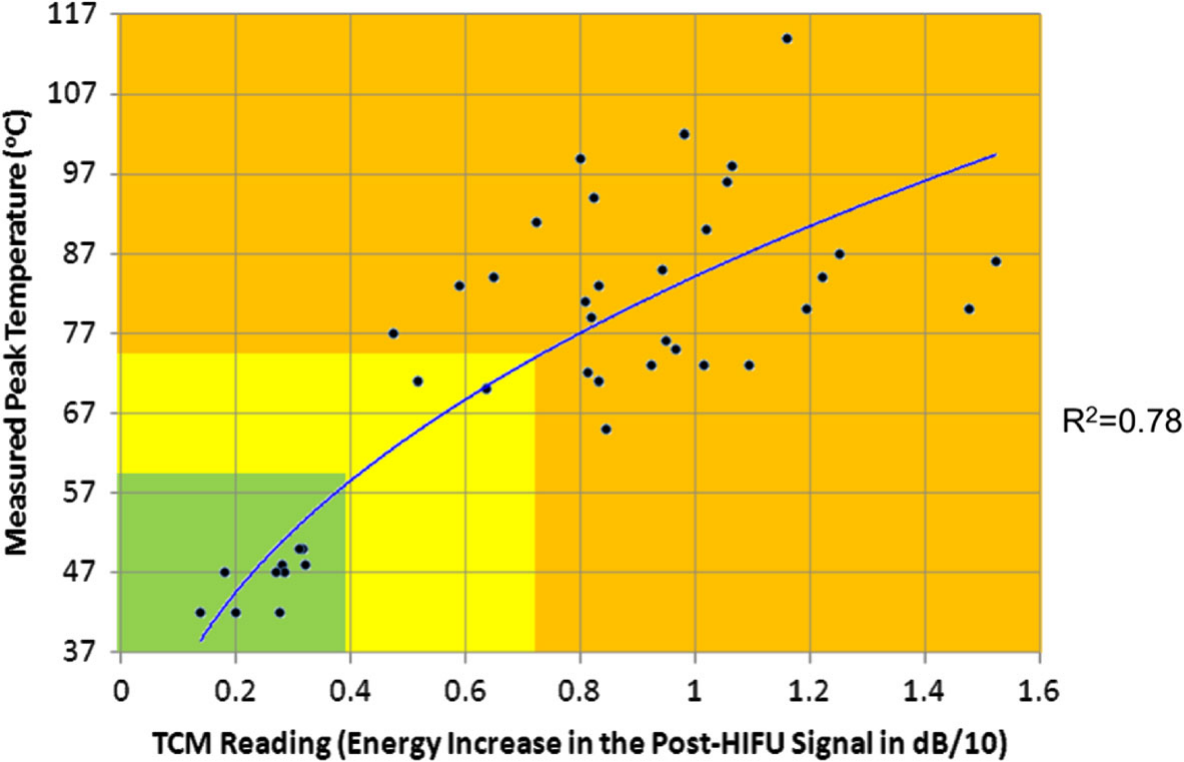
Calculated ΔE (dB/10) vs measured peak temperature in the prostate

The peak temperature and calculated ΔE values were derived for each ablative sites for these 5 patients. The cross correlation coefficient (R^2^) between a trend line and the dataset was 0.78 (Fig. 13). The false TCM detection rates were <5% for all patients.

During the study it was found that the TCM algorithm was not sensitive to normal pulsatile bowl motion; however it was sensitive to larger bodily movement that was caused intentionally by the operator to test TCM sensitivity. The TCM algorithm is sensitive to aberration of HIFU beam due to calculi in the HIFU path resulting in false TCM values. It was also found that the TCM algorithm was more prone to generating false TCM values during the last session of posterior prostate ablation. We hypothesize that this is due to nearfield heating caused during the previous anterior and middle zone ablations, which have contributed to raising over time the attenuation of the posterior prostate region (and associated reference values of SR(f)), resulting in a smaller dynamic range for the computation of the TCM. The prostate was allowed to cool in some cases prior to treating the posterior zone. The cooling of the prostate reduced false TCM results, providing supporting evidence to the hypothesis above. The TCM software provided user guidance to obtain B-mode images with optimized DGC and gain settings for the best TCM outcome.

## Discussion

The most important issue in thermal tissue ablation of prostate tissue by HIFU is to understand the delivery and control of energy exposure inside the tissue. When tissue temperature is maintained between 50 and 80 degrees Celsius (°C) for a few seconds (less than 5 s), tissue protein denaturation occurs, which results in tissue necrosis, which is also known as thermal lesion. At the low end of this range, the temperature must be maintained for several seconds to achieve tissue necrosis. At the high end of this range, necrosis occurs very rapidly [O′Brien et al. [24]]. For different tissue types thermal lesion thresholds are different and particularly for deep targeted tissues where ultrasound propagates thru several inhomogeneous layers that make predictable thermal lesion production technically challenging. Similarly, between patients and within the same prostate gland there are substantial differences in ultrasound absorption and attenuation coefficients due to tissue composition particulars. These differences can be seen in the transrectal ultrasound, CT and MRI images of the prostate and surrounding tissue [25]. Hence, to achieve reliable ablation of all targeted prostate tissues at each individual ablation site requires a reliable feedback mechanism. This feedback should allow for adjusting the HIFU energy delivery to compensate for the differences of absorption, attenuation and reflections of HIFU found throughout the prostate gland. This challenging feedback requirement has inspired numerous in vitro and in vivo studies to investigate tissue related ultrasound parameters as a quantitative predictor of tissue temperature reaching necrosis [26, 27]. For the TCM approach described in this report, several significant changes were required to implement the TCM algorithm in the Sonablate clinical device, including real-time, pulse-echo signal processing in the frequency domain, pre- and post-HIFU signal acquisitions for each individual site in the shortest time possible for a mechanically scanned transducer, the averaging of 15 pulse-echo lines, and optimizing the peak energy difference detection scheme using a two dimensional region of interest (ROI) located in the focal zone of the HIFU transducer [20]. The in vitro study provided a baseline on which to extend the TCM algorithm to clinical validation and verification. This approach was further fine-tuned during the Software validation clinical study of eight patients, as it provided in vivo pulse-echo data for algorithm optimization. During the In-Vivo Temperature Correlation to ΔE clinical study, a motion artifact was further introduced by pressing the super-pubic region of the patient, and its effect on TCM robustness was investigated. For these conditions, the TCM readings produced false positive results. Because of the good correlation between TCM and treatment outcome [23], TCM is now a tool that is part of all current Sonablate devices, providing the clinician additional feedback on which to make adjustments to the treatment plan to compensate for prostate tissue variations. Furthermore, the possibility to re-target and ablate the site that did not receive desired HIFU dose first time has enhanced the overall outcome of the whole gland prostate ablation without compromising safety.

## Conclusion

The TCM algorithm is able to estimate tissue changes reliably during the prostate tissue ablation during the HIFU procedure and that it can be used as a guide for HIFU dose delivery and tissue ablation control for the prostate tissue.

## Abbreviations

CE: Contrast enhanced
HIFU: High intensity focused ultrasound
MRI: Magnetic resonance imaging
PSA: Prostate specific antigen
ROI: Region of interest
TC: Thermocouple
TCM: Tissue change monitoring
US: Ultrasound

## References

[1] Foster RS, Bihrle R, Sanghvi NT, Fry FJ, Donohue JP (1993). High-Intensity Focused Ultrasound in the Treatment of Prostatic Disease. Eur Urol.

[2] Sanghvi NT, Fry FJ, Bihrle R, Foster RS, Phillips MH, Syrus J, Zaitsev AV, Hennige CW (1996). Noninvasive surgery of prostate tissue by high-intensity focused ultrasound. IEEE Trans Ultrason Ferroelectr Freq Control.

[3] Gelet A, Chapelon JY, Margonari J, Theillere Y, Gorry F, Souchon R, Bouvier R (1993). High-Intensity focused Ultrasound Experimentation on Human Benign Prostatic Hypertrophy. Eur Urol.

[4] Susani M, Madersbacher S, Kratzik C, Vingers L, Marberger M (1993). Morphology of Tissue Destruction Induced by Focused Ultrasound. Eur Urol.

[5] Madersbacher S, Pedevilla M, Vingers L, Susani M, Marberger M (1995). Effect of High-Intensity Focused Ultrasound on Human prostate Cancer *In Vivo*. Cancer Res.

[6] Gelet A, Chapelon JY, Bouvier R (1996). Treatment of prostate cancer with Transrectal focused ultrasound: early clinical experience. Eur Urol.

[7] Uchida T, Sanghvi NT, Gardner TA, Koch MO, Ishii D, Minei S, Satoh T, Hyodo T, Irie A, Baba S (2002). Transrectal high-intensity focused ultrasound for treatment of patients with stage T1b-2N0M0 localized prostate cancer: A preliminary report. Urology.

[8] Illing RO, Leslie TA, Kennedy JE, Calleary JG, Ogden CW, Emberton M (2006). Visually directed high-intensity focused ultrasound for organ-confined prostate cancer: a proposed standard for the conduct of therapy. BJU Int.

[9] Clarke RL, ter Haar GR (1996). Temperature rise recorded during lesion formation by high-intensity focused ultrasound. Ultrasound Med Biol.

[10] Damianou CA, Sanghvi NT, Fry FJ, Maass-Moreno R (1997). Dependence of ultrasonic attenuation and absorption in dog soft tissue on temperature and thermal dose. J Acoust Soc Am.

[11] Worthington AE, Tranchtenberg J, Sherar MD (2002). Ultrasound properties of human prostate tissue during heating. Ultrasound Med Biol.

[12] Seip R, Tavakkoli J, Dines KA, Wunderlich A, Sanghvi NT, Crum LA. Real-time detection of multiple lesions during high intensity focused ultrasound (HIFU) treatments. Proceedings of the International Symposium on Therapeutic Ultrasound. 2002 July:168–75.

[13] Clarke RL, Bush NL, ter Haar GR (2003). The changes in acoustic attenuation due to *in vitro* heating. Ultrasound Med Biol.

[14] Lizzi FL, Deng CX, Muratore R, Ketterling JA, Alam SK, Mikaelian S, Andrew MA, Crum LA, Vaezy S (2003). Radiation-force motion technique for monitoring HIFU exposures. 2nd Int Sym Therapeutic Ultrasound, Conference Proceedings 2002.

[15] Curiel L, Souchon R, Rouviere O, Gelet A, Chapelon JY (2005). Elastography for the follow-up of high-intensity focused ultrasound prostate cancer treatment: Initial comparison with MRI. Ultrasound Med Biol.

[16] Simon C, Van Baren P, Ebbini ES (1996). Two-dimensional temperature estimation using diagnostic ultrasound. IEEE Trans Ultrason Ferrooelecttr Freq Control.

[17] Pernot M, Tanter M, Bercoff J, Waters KR and Fink M.. Temperature Estimation Using Ultrasoinc Spatial Compound Imaging, IEEE Trans Ultrason Ferrooelecttr. Freq Contro, Vol. 51, No. 5, 606-615, May2004:15217237

[18] R. Seip, J. Tavakkoli, R. Carlson, A. Wunderlich, N. Sanghvi, K. Dines and T. Gardner. “High-Intensity Focused Ultrasound (HIFU) Multiple Lesion Imaging: Comparison of Detection Algorithms for Real-Time Treatment Control. IEEE Ultrasonics Symposium Proceedings, pp. 1395–1398. 2002.

[19] Tavakkoli J, Sanghvi NT, Frenkel V (2011). Ultrasound-guided HIFU and thermal ablation. Therapeutic Ultrasound, Mechanisms to Applications.

[20] Chen W, Carlson R, Weis C, Seip R, and Sanghvi NT. System and Method For Tissue Change Monitoring during HIFU treatment. US Patent 8,235-902 B2, August 7, 2012. (https://www.uspto.gov/patents-application-process/search-patents).

[21] Chen W, Carlson R, Sanghvi NT, Uchida T (2011). Rea-Time Tissue Change Monitoring on the Sonablate 500 during HIFU Treatment of Prostate Cancer. 10^th^ International Symposium on Therapeutic Ultrasound (ISTU) 2010. AIP Conf Proc.

[22] Investigational Device Exemption (IDE) # G000276 and G060129 –Treatment of T1/T2 Prostate Cancer by the Sonablate HIFU device, granted by the Food and Drug Administration. (https://clinicaltrials.gov/ct2/show/NCT00770822).

[23] Uchida T, Tomonaga T, Kim H, Nakano M, Shoji S, Nagata Y and Terachi T. Improved Outcomes with Advancements in High Intensity Focused Ultrasound Devices for the Treatment of Localized Prostate Cancer. The Jour of Urology, Vol. 193, 103-110, January 2015. 10.1016/j.juro.2014.07.096 25079940

[24] O’Brien WD, Deng CX, Harris GR, Herman BA, Merritt CR, Sanghvi NT, Zachary JF (2008). The risk of exposure to diagnostic ultrasound in postnatal subjects: thermal effects. J Ultrasound Med.

[25] Haas CA and Resnick MI. Imaging of the Prostate. Chapter 6, Published in “Prostatic Diseases”, Edited by Herbert Lepor; MD. W. B. Saunders Company, ISBN 0–7–7216-7416-X.

[26] Rahimian S, Tavakkoli J (2013). Estimating dynamic changes of tissue attenuation coefficient during high-intensity focused ultrasound treatment. Journal of Therapeutic Ultrasound.

[27] Vaezy S, Shi X, Martin RW, Chi E, Nelson PI, Bailey MR, Crum LA (2001). Real-time visualization of high-intensity focused ultrasound treatment using ultrasound imaging. Ultrasound Med Biol.

